# Species Sorting and Neutral Theory Analyses Reveal Archaeal and Bacterial Communities Are Assembled Differently in Hot Springs

**DOI:** 10.3389/fbioe.2020.00464

**Published:** 2020-05-28

**Authors:** Lianwei Li, Zhanshan (Sam) Ma

**Affiliations:** ^1^Computational Biology and Medical Ecology Lab, State Key Laboratory of Genetic Resources and Evolution, Kunming Institute of Zoology, Chinese Academy of Sciences, Kunming, China; ^2^Kunming College of Life Science, University of Chinese Academy of Sciences, Kunming, China; ^3^Center for Excellence in Animal Evolution and Genetics, Chinese Academy of Sciences, Kunming, China

**Keywords:** hot spring microbiome, community assembly, species sorting, neutral theory, archaea, bacteria

## Abstract

Although the recognition of archaea as one of the three kingdoms in the tree of life has been nearly a half-century long, the comparative investigations on their ecological adaptations with bacteria have been limited. The mechanisms of their community assembly and diversity maintenance in hot springs have not been addressed. The mechanistic study is critical not only for understanding the hot-spring microbiome structure and dynamics, but also for shedding light on their evolutionary adaptations. We applied the neutral theory model and species sorting paradigm of metacommunity theory to investigate how hot-spring microbial communities were assembled, how their diversities were maintained, and how the temperature and pH influence these mechanisms. Through rigorous statistical tests based on the neutral theory and species sorting paradigm, we found (*i*) According to the neutral theory, archaeal and bacterial communities are assembled differently, with stochastic neutral force playing a more significant role in archaeal communities than in bacterial communities (neutrality-rate = 52.9 vs. 15.8%, *p*-value < 0.05). (*ii*) Temperature and pH account for rather limited (<10%) variations in hot-spring microbiomes based on the species sorting paradigm. The pH has more significant influences than temperature on archaeal communities, and both pH and temperature have similarly low influences on bacterial community structure. (*iii*) We postulate that the differences between archaea and bacteria are likely due to the longer evolutionary history and better adaptation of archaea to host spring environments.

## Introduction

Hot springs are natural water environments present all over the world. The diversity and stability of the microbial communities in hot springs are influenced by the geothermal environment they distribute (e.g., Chan et al., 2017; Ghilamicael et al., 2017; Poddar and Das, 2018). Microorganisms, as the basis of food web, play an important role in energy metabolism and matter cycling of these thermal systems (Cai et al., 2013; Lin et al., 2015). For example, carbon assimilatory pathways are one of the most important metabolic pathways for hot springs. Hot springs can be characterized by several specific physical and chemical characteristics, including temperature, pH and chemical components. These physicochemical properties are the main factors affecting the microbial components in hot spring ecosystems (Chan et al., 2017; Ghilamicael et al., 2017; Poddar and Das, 2018). In general, microbial diversity is negative correlated with temperature of hot spring (Miller et al., 2009; Cole et al., 2013), but some exceptions have been reported (Wang et al., 2013). Although, in general, temperature is the most important factor shaping the structure of thermal spring microbial communities, the role of pH cannot be ignored either (Inskeep et al., 2013a, b; Song et al., 2013; Xie et al., 2015; Jiang and Takacs-Vesbach, 2017). For instance, the pH of Yellowstone National Park (YNP) thermal springs ranged between 1 and 10, and were classified into acidic, vapor-dominated and alkaline, water-dominated two systems. Studies also found that the taxa in *Aquificales*, *Chloroflexi*, and *Cyanobacteria* dominate the microbial communities in alkaline springs whereas Archaea predominate the microbiota in acidic springs (Inskeep et al., 2013a, b; Jiang and Takacs-Vesbach, 2017). Chemical types of hot springs may also affect the organization of microbial community. In acid sulfate hot springs with low chloride, there are significant differences of the diversity and composition structure between the microbial communities in upstream and downstream outflow channel region, which are associated with the total organic carbon, dissolved oxygen and accumulated arsenic (Jiang et al., 2015).

In spite of the extensive studies on the hot spring microbiome ecology in recent years (Li and Ma, 2019a,b,c; Li et al., 2020), to the best of our knowledge, the underlying mechanisms that shape the hot spring microbiome structure and dynamics, or community assembly and diversity maintenance in the terminology of theoretical community ecology, have not been investigated. Traditionally, with cross-sectional community observational datasets, there are two ecological theories that can be harnessed to discern the mechanisms we are interested in, i.e., the neutral theory and niche theory (Ferrenberg et al., 2013; Lee et al., 2013; Szekely and Langenheder, 2013; Zhang et al., 2014; Liao et al., 2016; Langenheder et al., 2017; Cira et al., 2018; Tripathi et al., 2018). Hubbell’s (2001, 2006) neutral theory of biodiversity, which was inspired by the neutral theory of molecular evolution, is the most well-known neutral model, although there are several other versions of neutral theory models, which do not make specific assumption on species traits or their responses to the environment (Chave, 2004; Chase, 2005; Chase et al., 2005; Chase and Bengtsson, 2011). The neutral theory hypothesizes that species are neutral regarding their inter-specific interactions as well as the underlying environment, which implies that the numbers of individuals and species in any given locality are govern by stochastic processes (Alonso et al., 2006; McGill et al., 2007; Volkov et al., 2007; Rosindell et al., 2011). Although the assumptions of the neutral theory model may not be realistic in natural communities, the test of neutrality, which compare the observed species abundance distribution (SAD) with the predicted species relative abundances from the neutral theory model, can highlight the effect of stochastic processes and can determine how far one may go by making the simplest assumption—communities are assembled by stochastic processes only. On the other hand, if the neutrality test fails, it also indicates the existence of non-stochastic processes (deterministic processes) such as species sorting based on niche differentiations are in effect in the community. In this study, we take advantage of this complementary aspect with species sorting framework to infer the true mechanisms underlying the community assembly and diversity maintenance in hot spring microbiomes.

Contrary to the assumptions of the neutral theory, species sorting, as one of the most commonly used frameworks for implementing the niche theory, ignores the effects of stochastic processes and instead focuses on the deterministic processes that result from different responses of species to heterogeneous environments such as water temperatures and pH in the case of hot spring microbiomes. In other words, different spectrums of environments represent different niches for different species, i.e., the niche differentiation (Chase and Leibold, 2003; Chase and Bengtsson, 2011; Adams et al., 2014). According to species sorting theory, a species will occur in a locality if the environments, which can be biotic or abiotic, are favorable. In other words, species self-assemble into clusters based on their niche requirements. Trade-offs in adapting to different environments such as temperatures or pH in our case, can be invoked to indicate that species are favored under some environments will be disfavored under others (Chase and Bengtsson, 2011).

As emphasized by Chase and Bengtsson (2011) rightly, in any metacommunity (i.e., a collection of local communities, such as the 165 hot springs distributed globally, whose microbial communities were sampled and to be analyzed in this study), a variety of processes including both stochastic and deterministic forces are usually in effect simultaneously (Chase, 2005; Chase et al., 2005; Holyoak et al., 2005; Ferrenberg et al., 2013; Lee et al., 2013; Liao et al., 2016; Langenheder et al., 2017; Cira et al., 2018). The challenge is to disentangle the relative significance of the different processes. In particular, the focus should be centered on identifying features of a given environment type, that would allow for one or the other set of processes exert a stronger influence on community patterns (Chase and Bengtsson, 2011). This is also one of the two objectives of the present study, i.e., identifying the influence of temperatures and pH on the processes that governs the community assembly and diversity maintenance in hot spring microbiomes. Furthermore, we have a second objective, which is closely intermingled with the first objective, of determining the role of stochastic neutral processes in driving the community assembly and diversity maintenance.

The two objectives we pursue, if achieved successfully, are of important theoretical and practical significances. Theoretically, the complementary analyses with species sorting and neutral theory touch a fundamental question in natural ecosystems. How do species coexist and form a diverse life world, rather than go extinct or the earth is dominated by a single species, alternatively? It was Darwin who first posed the question in the epilog (last paragraph) of his “On the Origin of Species” by contemplating “an entangled bank,” which is a metaphor of ecological community in the modern terms. Obviously, both Darwin’s evolutionary theory and metacommunity theory tackled a common question, why the life is so diverse and how species coexist. Indeed, Darwin’s “struggle for life” emphasized the competition, which intuitively would lead to competitive exclusions, and therefore science dearly needed theories that can explain the obvious coexistence and diversity in nature. This has been the case since Darwin’s evolutionary theory was first put forward and metacommunity theory is just one of the most important attempts by ecologists in the turn of the new century. Practically, we hope our study will help to determine if there exist fundamental differences between archaea and bacteria in terms of their ecological adaptations to the hot spring environment. This question is important because, although archaea and bacteria have been taxonomically classified as two independent kingdoms in the three-kingdom life tree for nearly a half-century (Eme et al., 2017), the investigation on their ecological adaptations from metacommunity theory perspective has not been conducted to the best of our knowledge.

## Materials and Methods

### Datasets of the Hot-Spring Microbiomes

The datasets of microbiome samples from 165 hot springs were originally collected and reported by Sharp et al. (2014). Their 16S-rRNA OTU (operational taxonomic unit) tables were generated from the 165 microbiome samples taken from sediment, soil and mat in Western Canada and Taupo Volcanic Zone, New Zealand (Sharp et al., 2014). A total of 1,162,553 high quality sequences were obtained, from which 61,910 OTUs, including 7,964 archaea and 53,946 bacteria, were clustered at the 97% similarity level (species level). Further information on the datasets is referred to Sharp et al. (2014).

The object of Sharp et al. (2014) was to test for a temperature-diversity gradient in geothermal environments covering a much broader temperature range. The temperatures ranged 7.5–99°C, and pH-values ranged 1.8–9.0 in those 165 hot spring samples. They evaluated the relative influences of temperature and pH on microbial diversity and tested whether temperature has a direct effect on diversity in ecosystems. They found that temperature had the greater influence than pH on OTU richness and Shannon diversity. Sharp et al. (2014) datasets also offer excellent opportunities to investigate the mechanisms of community assembly and diversity maintenance in hot springs, especially with such wide ranges of temperatures and pH. We therefore apply the metacommunity models of species sorting and neutral theory to reanalyze the datasets to take advantages of the opportunities.

### Species Sorting Model

Species sorting analysis is built on theories of community change over environmental gradients and it considers the effects of local abiotic factors on population demographic rates and species interactions. Accordingly, local patches are considered as heterogeneous in some factors and the outcome of local species interactions depends on aspects of the abiotic environment.

When different species can only inhabit exclusive habitat types, the resulting metacommunity can be clustered into two independent groups (Leibold et al., 2004). However, if individual species can inhabit multiple habitat types, there will be a variety of outcomes that reflect how species interact at larger spatial scales. One strategy to model such dynamics is then to extend assembly models to systems with multiple patch types (Leibold et al., 2004), such as Tang and Zhou’s (2013) hybrid model.

Species sorting analysis aims to determine the influence of environmental factors (pH and temperature in the case of hot springs of this study) on the composition and assembly of ecological communities. We utilized the RDA function in the Vegan R-package (Livingston et al., 2012) to perform the redundancy analysis (RDA), which quantifies the effect of each environmental factor as well as their combinations on the population abundance of each species in the community (Legendre and Legendre, 2012). The analysis is essentially a variance decomposition method that partitions total variance of the OTU matrix into various portions explainable by the environmental factors.

RDA (redundancy analysis) is one kind of canonical analysis, which allows for simultaneous analysis of two or even several data tables (matrixes). Therefore, it is possible to perform a direct comparison of two data matrices such as the matrix of OTU abundances (tables) and the matrix of environmental factors (pH and temperatures). The so-termed canonical form refers to the simplest and most comprehensive form to which certain functions, relations, or expression can be reduced without loss of generality. Canonical analysis integrates the concepts of ordination and regression, which involves two matrices: a response matrix **Y** (e.g., OTU table) and an explanatory matrix **X** (e.g., pH and temperature data). On the one hand, the analysis usually generates orthogonal axes from which scatter diagrams can be plotted (so-termed biplot) to display the principal (canonical) ordinates and the effect of explanatory variable **X** on response matrix **Y**. On the other hand, RDA is the direct extension of multivariate regression to the modeling of multivariate response data.

The redundancy measure the explained variance, and the results of RDA indicate the change (variance) of **Y** explained by the canonical ordinates. With RDA, each canonical ordination axis, similar to a principal component, corresponds to a direction, in the multivariate scatter of objects (matrix **Y**). The canonical ordination is maximally related to a linear combination of the explanatory variables **X** (Legendre and Legendre, 2012).

### Hubbell’s Neutral Theory of Biodiversity

There have been quite a few applications of Hubbell’s (2001) neutral theory to microbial communities in recent years (Sloan et al., 2006, 2007; Woodcock et al., 2007; Holmes et al., 2012; Jeraldo et al., 2012; Levy and Borenstein, 2013; Morris et al., 2013; Nemergut et al., 2013; Barberán et al., 2014; Harris et al., 2015; Venkataraman et al., 2015; Roguet et al., 2015; Zeng et al., 2015; Chen et al., 2017; Dai et al., 2017). Hubbell’s (2001) neutral theory is an individual-based sampling theory, and it offers a biological occurrence mechanism to interpret the observed SAD in an ecological community. It assumes that all individuals in a saturated local community are ecologically equivalent, i.e., they have the same birth, death and migration rates, excluding their random fluctuations (Li et al., 2018). Here we use Etienne (2005, 2007) sampling formulae and “Exact” test of neutrality, i.e., the statistical procedures to fit the neutral theory model and perform statistical tests between the model prediction and the actual SAD presented in the microbiome datasets, i.e., the OTU table or **Y** used for species sorting analysis introduced in the previous section. In the neutrality test, the explanatory matrix **X** (environmental factors) in previous species sorting does not play a role because the responses of species to different environmental gradients are assumed to be neutral (making no differences).

The Etienne’s “Exact” neutrality test is based on the sequential construction schemes, and it does not require alternative model in hypothesis testing. Hence, it avoids the discussion of validity of the alternative model in empirical evaluations (Etienne, 2007), which is an advantage because there can be many alternative SAD models such as the lognormal, log-series and power law distributions. The detailed computational procedures for implementing the neutrality test of Hubbell’s neutral theory with the maximum likelihood estimation (MLE) and log-likelihood ratio (LLR) test are referred to Etienne (2005, 2007) and Li and Ma (2016). The code for testing the neutral theory is from the supplementary information of Etienne (2005). The *p*-value of 0.05 (*p* > 0.05) is adopted as the threshold for passing the neutrality test.

## Results and Discussion

### The Neutral Model

**FIGURE 1:**
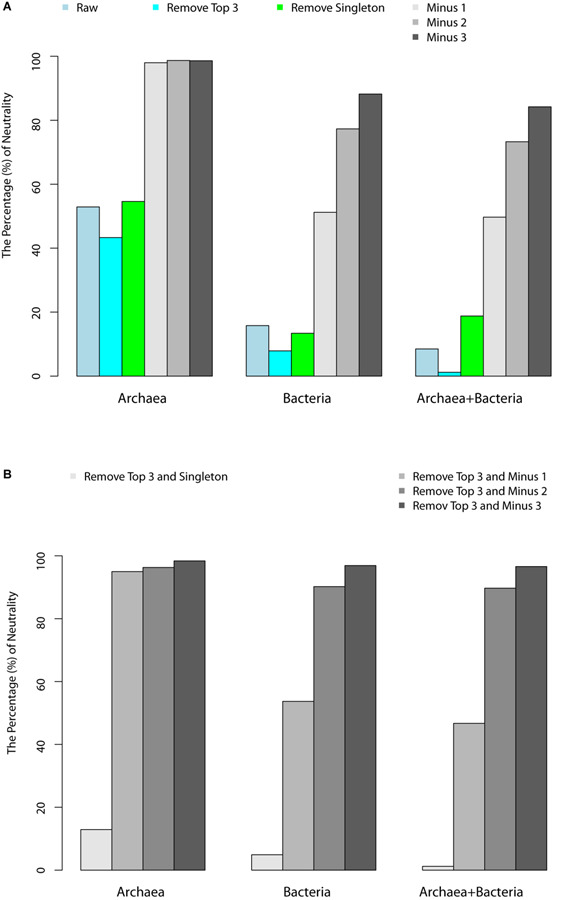
**(A)** Percentages of hot-spring microbiome samples that passed the neutrality test with the schemes of “remove top 3,” “remove singleton,” “minus 1,” “minus 2,” and “minus 3.” **(B)** Percentage of samples that passed the neutrality test with “remove top 3 and singleton,” “remove top 3 and minus 1,” “remove top3 and minus 2,” “remove top 3 and minus 3.”

**FIGURE 2:**
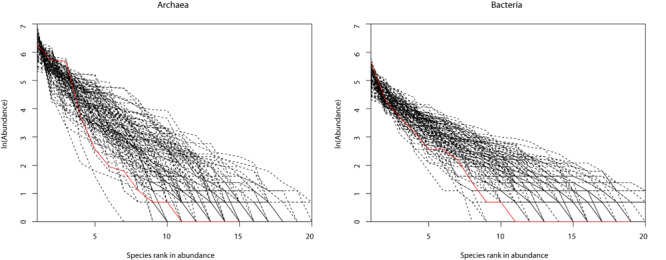
Two examples of the hot-spring microbiome samples that passed the neutrality test: **(A)** archaea and **(B)** bacteria.

Table 1 (also see Figures 1A,B) shows that slightly more than 1/2 of the archaea communities satisfied the prediction of the neutral theory model. Approximately 16% of bacteria community satisfied the neutral theory model (Column 2). We postulated that rare species and/or dominant species might be responsible for the lack of neutrality in the hot spring microbiome. We therefore designed three additional test schemes: remove the top three most abundant species; remove singleton or minus 1, 2, or 3 individuals, from each community sample; and the combination of the first two schemes, i.e., remove the top three most dominant and simultaneously removing singleton, or 1, 2, or 3 individuals, respectively. Figure 2 shows two example of successful fitting to the neutral theory model, one for archaea and the other for bacteria.

**TABLE 1:** The percentages (%) of hot-spring-microbiome community samples that passed the neutrality test with four different test schemes [*p*-value adjusted with standard FDR control (Benjamini and Hochberg, 1995)].

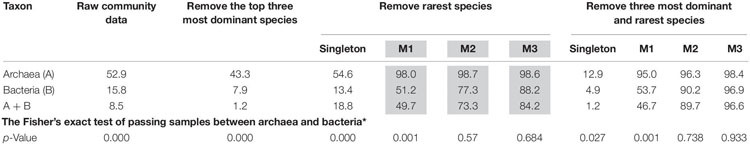

*If *p*-value < 0.05, the neutrality passing rates are significantly different between archaea and bacteria.

We summarize the findings from the three regimes as follows:

(i) Archaea communities are more likely to pass the neutrality test, given that slightly more than 1/2 of the samples passed the test without adopting any of the pre-processing removal of rare or dominant species (i.e., with raw datasets). Bacterial communities are far less likely (only 16%) to pass the neutrality test and the combined (mixed) community of archaea and bacteria failed to pass the test in most cases (only 8.5%).
(ii) Removal of the top three most abundant species or single does not improve the fitting of neutral theory model. In fact, it actually lowered the goodness-of-fitting of the neutral model. Removal of singleton does not have significant impact on the performance of neutral theory model, except for the combined dataset of archaea and bacteria.
(iii) Removal of one, two, or three individuals from each species in the community, respectively, led to dramatic improvement of the neutral-model fitting. Removal of one individual across all species resulted in the neutrality passing rates of 98, 51.2, and 49.7% corresponding to archaea, bacteria and their combined, respectively; removal of two individuals resulted in 98.7, 77.3, and 73.3%; removal of three individuals resulted in 98.6, 88.2, and 84.2%, for archaea, bacteria, and their combined, respectively.
(iv) We further analyzed the relationship between the pass/fail (*p*-value) of neutrality test and pH or temperature. Figures 1A,B showed that most failed cases (*p* < 0.05) occurred in high pH or low temperature range, when three individuals were removed from each species. This seems to suggest that the effect of temperature on community assembly was in the lower temperature range, while that the effect of pH on community assembly was in the high pH range. In other words, the selective forces were more likely in effect at lower temperatures and high pH values. The underlying mechanism for this observation is not clear to us.
(v) The effect from removal of both dominant and rare species in terms of passing the neutrality test was largely from the removal of rare species, as shown in Table 1.
(vi) The effect of removal of one, two, or three individuals universally (across all species) is similar to adjustment of the system errors in instrument measurement. In the case of 16S-rRNA sequencing, it should be equivalent to adjusting the sequencing errors. Therefore, we cautiously conclude that archaea communities are largely driven by the neutral process (force), the effect of neutral processes in bacteria and the combined community of archaea and bacteria cannot be ignored either.

### Species Sorting Model

**FIGURE 3:**
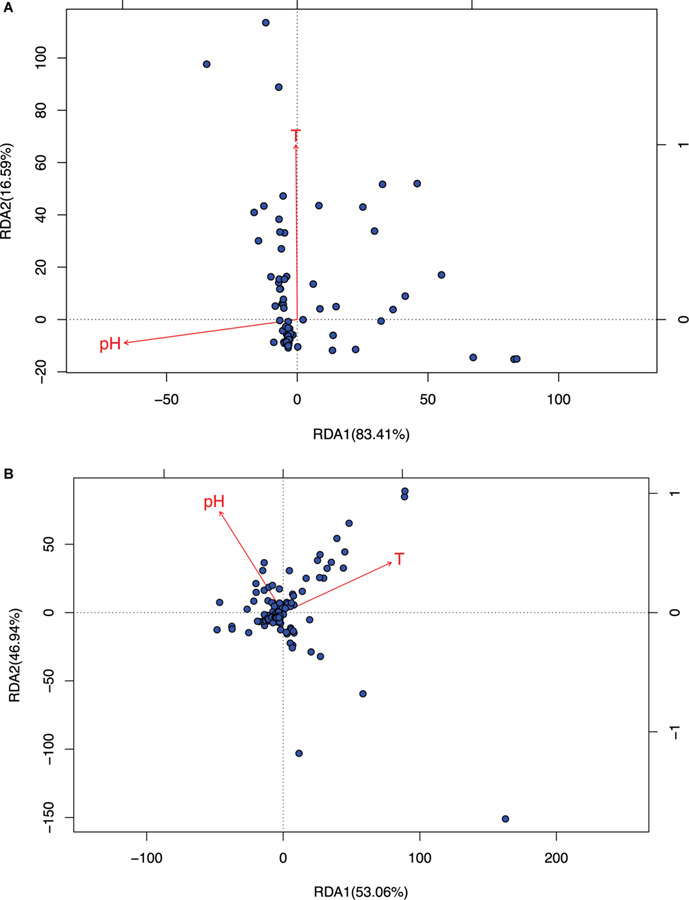
The RDA ordination biplot for the Archaea communities. **(A)** For archaea and **(B)** for bacteria: (*i*) Legends: black dots in the graph represent for the hot-spring microbiome samples, full-line arrows for the biplot scores of the environmental variables. (*ii*) Most sample points were clustered around the origin, and some scattered far from the origin, which may be significantly different from the samples clustered around the origin. (*iii*) The angular between pH and temperature is slightly larger than π/2, which suggests that they may be negatively correlated weakly. (i*v*) RDA1 and RDA2 are the linear combination of pH and temperature. In **(A)**, temperature and RDA1 are nearly perpendicular to each other, which suggested that temperature does not have an explanatory power for RDA1, and RDA1 is primarily explained by pH (pH and RDA1 is negatively correlated). The effect of temperature is primarily represented in RDA2, but RDA2 only weights 16.6% and therefore, in archaea, pH is the main factor to influence the community and temperature has little effect. In **(B)**, pH and temperature have similar explanatory power to both RDA1 and RDA2, that is, pH and temperature have similar level of influence to bacteria.

**FIGURE 4:**
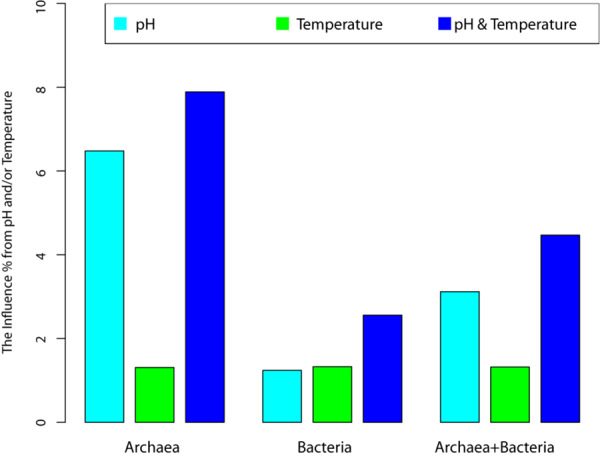
The variance of community changes explained by the environmental factors (pH, temperatures, and their joint effects).

To test the effect of environmental factors (pH and temperature) on community assembly and diversity maintenance, we performed RDA analysis with R-Package Vegan, which was designed to test the metacommunity model of species sorting. The results were listed in Table 2, and organized based on the same four test schemes utilized for neutrality test as performed in the previous section (i.e., raw sequencing data, removal top three most abundant species, remove singleton or minus 1, 2, or 3 across all species, and the combinations of the second and third schemes. From Table 2 (*also see* Figures 3, 4), we summarize the following findings:

**TABLE 2:** Species sorting analysis: the percentage of the influence of pH and/or temperature.

**Data preprocessing scheme**	**Communities**	**Environmental factors**			**RDA (canonical ordinates)**	
		**pH (%)**	**T (%)**	**pH and T (%)**	**RDA1 (%)**	**RDA2 (%)**
Raw community	Archaea (A)	6.48	1.31	7.89	83.4	16.6
	Bacteria (B)	1.24	1.33	2.56	53.1	46.9
	Archaea + Bacteria (A + B)	3.12	1.32	4.47	70.4	29.6
Remove singleton	Archaea (A)	6.48	1.31	7.88	83.4	16.6
	Bacteria (B)	1.24	1.32	2.55	53.1	46.9
	Archaea + Bacteria (A + B)	3.12	1.32	4.46	70.4	29.6
M1 = Minus *1* across all species	Archaea (A)	6.47	1.3	7.87	83.4	16.6
	Bacteria (B)	1.24	1.32	2.54	53	47
	Archaea + Bacteria (A + B)	3.11	1.31	4.45	70.5	29.5
M2 = Minus *2* across all species	Archaea (A)	6.46	1.3	7.86	83.5	16.5
	Bacteria (B)	1.23	1.32	2.54	53	47
	Archaea + Bacteria (A + B)	3.11	1.31	4.44	70.5	29.5
M3 = Minus *3* across all species	Archaea (A)	6.46	1.3	7.84	83.5	16.5
	Bacteria (B)	1.23	1.31	2.53	53.1	46.9
	Archaea + Bacteria (A + B)	3.11	1.31	4.44	70.5	29.5

(i) As shown in Table 2, it turned out that the four test schemes made no differences regarding the results of species sorting analysis. This should be somewhat expected because species sorting analysis focuses on general trend, rather than on minute details, as in the case of neutrality test. Figure 3 (*see* its legend) presented more detailed information about how the results from species sorting analysis should be interpreted.
(ii) Between the two canonical ordinates, RDA1 and RDA2, which are the linear combinations of the explanatory variables (the temperature and pH), RDA1 is far more important than RDA2 (83.4 vs. 16.6) in representing the community patterns in the case of archaea. But in the case of bacteria, both RDA1 and RDA2 are rather close with each other (53.1 vs. 46.9%). For the mixed community of archaea and bacteria, RDA1 is again more important than RDA2, but the difference in the importance level is between the differences in the case of archaea and bacteria alone.
(iii) The most important metrics from RDA analysis are the percentages associated with the environmental factors and/or their combinations. These percentages represent for their respective explanatory power (or contributory effect) to the canonical ordinates (RDAs). In the case of archaea, pH alone, T alone and their combination (pH & T) explained 6.48, 1.31, and 7.89% of the community change (variance), respectively. Hence, pH is much more important than temperature to the changes in archaeal communities, although the effect of either is rather limited. Even the combined explanatory power of temperature and pH is only 7.89%. For bacteria, both pH and temperature have similarly small explanatory power (under 2%), and their combined explanatory power is only 2.56%.
(iv) For the mixed community of archaea and bacteria, pH has twice more the explanatory power than temperature (3.12 vs. 1.32%), and the combined effect of pH and temperature only possess 4.47% explanatory power. Most likely, the relatively large explanatory power of pH is due to its larger influence on archaea in the mixed community.
(v) In general and in all cases, the effects of environmental factors (pH, temperature or their combined effects) are under 10%. That is, less than 10% variance of OTU abundance changes can be explained by the environmental factors. The breakup for archaea and bacteria as well as their mixed communities were illustrated in Figure 3 (also see Table 2). This finding confirmed our previous conclusion that the neutral force should play a significant role in archaea community and the role in bacterial community or the mixed community of archaea and bacteria cannot be ignore either.

We further computed the Spearman’s correlation coefficient between pH and population abundance *or* between temperature and population abundance. There were 782 (9.8%) archaea and 2733 (5.1%) bacteria species correlated significantly to pH, respectively. There were 916 (11.5%) archaea and 1512 (2.8%) bacteria species correlated significantly to pH, respectively. Furthermore, the absolute values of all the correlation coefficients were under 0.5, even for those passing statistical significance level (*p* = 0.05). This again confirms the limited influence of pH and temperature on community structure.

## Conclusion

The Arachea was recognized as a third domain, independent of the Bacteria approximately a half century ago (Eme et al., 2017). Nevertheless, whether their communities are assembled differently with those of bacteria has not been investigated previously. In the present study, through statistically rigorous tests, supported by several schemes for calibrating sequencing reads errors, it was demonstrated that the archaeal communities are three times more likely assembled by the stochastic neutral process than the bacterial communities are. In other words, the community assembly and diversity maintenance of archaeal communities are less likely influenced by the environmental factors (pH and temperature, revealed in this study) than those of bacterial communities. We postulate that this difference between archaea and bacteria may be due to the longer evolutionary history and consequently better adaptation of archaea to hot spring environments.

The complementary results from species sorting analysis revealed than pH, temperature or their combined effects explained less than 10% (1.24–7.89%) of the variations occurred in the SAD (in the form of OTU tables) among different hot spring microbiome samples. In other words, environmental factors have rather limited influences on the community structures of hot spring microbiomes. This cross-verified our finding from the neutral theory testing because it suggests that there should be a significant role for stochastic forces to play. The pH and temperature are well-recognized as two of the three most influential environmental factors (the other is chemicals in hot spring water), which controls much of the stability of the hot spring environment and therefore and exerts far reaching influences on the hot spring microbiome diversity and compositions (Mohanrao et al., 2016; Amin et al., 2017; Chan et al., 2017; Ghilamicael et al., 2017; Poddar and Das, 2018). Existing studies have suggested that there is an inversely proportional relationship between microbial diversity and temperature of hot spring (Cole et al., 2013; De León et al., 2013; Amin et al., 2017; Chan et al., 2017). In addition to temperature, water pH is another primary environmental factor directly influencing microbial diversity in hot springs (Inskeep et al., 2013a, b; Xie et al., 2015). Given the critical importance of pH and temperature to hot spring ecosystems, our findings should have general validity and we postulate that it is unlikely that the chemical can explain the remaining 90% of the variation. That means, stochastic neutral forces do play an important role in the hot spring microbiome assembly and diversity maintenance.

Although there is currently a lack of comprehensive datasets on hot spring chemicals prevented us from quantifying its influence on the microbiome, the influence of chemical is more likely less than the combined effect of pH and temperature. This is because, the influence of chemicals in hot springs on microbes is mostly indirect, through their changes on pH, and therefore, likely has already been, at least partially, factored in our previous analysis with species sorting. For example, the water pH is determined by the chemical components in hot springs (Jiang et al., 2015; Geesey et al., 2016). Nevertheless, future studies that incorporate the influences of spring chemicals should be useful for further validating our findings presented in this study. Another issue we could not address in the present study is the relationship between bacterial quorum sensing and community assembly, which is of critical theoretic and practical significances. This is primarily because both species sorting and neutral theory were initially developed in macrobial ecology of plants and animals, in which the phenomenon of quorum sensing does not exist. In addition, the lack of longitudinal samples at the scale of quorum sensing also prevented us from exploring the role of quorum sensing in driving community assembly and maintaining diversity.

In conclusion, our study revealed that archaeal and bacterial communities are assembled differently, with stochastic neutral force playing a more significant role in archaeal communities than in bacterial communities. The neutrality rate (percentage of microbiome samples passing the neutrality test) in archaea is three times higher than in bacteria. That is, compared with more ancient archaea, bacteria are evolved under stronger selection forces. Nevertheless, temperature and pH account for rather limited (<10%) variations in hot-spring microbiomes based on the species sorting paradigm. This suggests that additional yet unknown selection forces may play important role in shaping the community assembly and diversity maintenance in the hot-spring microbiomes.

## Data Availability Statement

The hot-spring microbiome dataset is available at Sharp et al. (2014) publication site.

## Author Contributions

ZM designed the study, interpreted the results, and wrote the manuscript. LL conducted the computation and participated in the result interpretation. All authors read the manuscript and approved the submission.

## Conflict of Interest

The authors declare that the research was conducted in the absence of any commercial or financial relationships that could be construed as a potential conflict of interest.
